# Non-invasive respiratory support attenuates lung damage in a murine model of bronchopulmonary dysplasia

**DOI:** 10.3389/fped.2026.1755113

**Published:** 2026-04-20

**Authors:** Jitendra K. Tripathi, Anh Duong, Changgong Li, Parviz Minoo, Beiyun Zhou, Inderpal Randhawa, Nathan L. Marsteller

**Affiliations:** 1 Translational Pulmonary and Immunology Research Center, Long Beach, CA, United States; 2 Division of Neonatology, Department of Pediatrics, LAC+USC Medical Center and Childrens Hospital Los Angeles, Los Angeles, CA, United States; 3 Hastings Center for Pulmonary Research, Keck School of Medicine of University of Southern California, Los Angeles, CA, United States; 4 Division of Pulmonary, Critical Care and Sleep Medicine, Department of Medicine, Keck School of Medicine, University of Southern California, Los Angeles, CA, United States; 5 Pediatric Pulmonology Division, Memorial Care Health System, Long Beach, CA, United States

**Keywords:** bronchopulmonary dysplasia (BPD), continuous positive airway pressure (CPAP), hyperoxia, hyperoxia mouse model, mouse pulmonary function test, non-invasive respiratory support

## Abstract

Bronchopulmonary dysplasia (BPD) is a chronic lung disease primarily affecting preterm infants exposed to prolonged oxygen therapy and mechanical ventilation. This study investigates the potential effects of a non-invasive respiratory support strategy, Continuous Positive Airway Pressure (CPAP) on lung function and histopathological changes in a hyperoxia-induced murine model of BPD. The aim is to determine whether non-invasive respiratory support can attenuate lung damage caused by hyperoxia. In this study, mice were exposed to hyperoxia (60% O_2_) and treated with non-invasive respiratory support. Histopathological analysis was conducted to evaluate alveolar simplification and septal thickening to assess structural injury and repair. Lung function was evaluated using key parameters including static lung compliance (Cst), airway resistance (Rrs), respiratory system elastance (Ers), and other parameters to assess mechanical properties. Additionally, the experimental findings indicate that non-invasive respiratory support treatment produced a robust preservation of lung function, with strong maintenance of compliance (Cst; *p* = 0.0416) and marked reductions in airway elastance (Ers; *p* = 0.0022) and resistance. Histological analyses revealed substantial attenuation of alveolar injury (MLI; *p* = 0.0028), clear reduction in septal thickening, and pronounced suppression of pathological structural remodeling (septal thickness; *p* < 0.0001) in treated mice compared with hyperoxia-exposed controls (60% O₂).These results indicate non-invasive respiratory support not only enhances pulmonary mechanics but also mitigates hyperoxia-induced structural damage, offering insights into its potential application in managing BPD in preterm infants. This study highlights the significance of non-invasive ventilation strategies in reducing the severity of BPD and potentially improving outcomes.

## 1 Introduction

Bronchopulmonary Dysplasia (BPD) is a chronic lung disease primarily affecting premature infants who require prolonged mechanical ventilation and oxygen therapy due to respiratory distress syndrome (RDS). It is characterized by impaired alveolar development, persistent inflammation, and fibrosis, leading to long-term respiratory problems (1, 2). BPD affects approximately 10,000–15,000 infants per year in the U.S., with a reported incidence ranging from 20% to 45% in very low birth weight (VLBW) infants (<1,500 g) or those born before 30 weeks of gestation (3). While BPD rates are similar in countries with advanced neonatal care, the incidence may be lower in low-resource settings due to decreased survival of preterm infants requiring intensive care. Global studies indicate an incidence of around 40% in infants born before 29 weeks of gestation (4). However, the reported BPD incidence varies widely across studies, mainly due to inconsistent diagnostic criteria. Definitions differ by assessment timepoint (28 days vs. 36 weeks PMA) and by thresholds for oxygen or respiratory support. These variations directly affect which infants are classified as BPD, leading to substantial differences in reported incidence rates.

The etiology of BPD is multifactorial, involving a combination of prematurity, oxygen toxicity, mechanical ventilation, and infection/inflammation (5). The key issue in BPD is the disruption of alveolarization, the process by which alveoli form and mature. Under normal circumstances, late gestation is marked by rapid alveolar development, increasing the surface area available for gas exchange (6).

Emerging evidence suggests alveolarization does not proceed as a simple linear continuum, but rather as a developmentally gated program that can be suppressed or re-engaged depending on environmental and mechanical cues. Hyperoxia represents a significant inhibitory signal, while controlled distending pressure may preserve or partially restore septation-associated programs during vulnerable developmental windows. Understanding how these competing inputs interact is critical to defining whether impaired alveolarization reflects irreversible arrest or context-dependent modulation.

Alveolarization is a critical phase of post-saccular lung development that establishes the structural basis for efficient pulmonary gas exchange. This process involves secondary septation of distal saccules into mature alveoli, progressive thinning of alveolar septal walls, and coordinated development of the pulmonary microvasculature. In humans, alveolarization begins near term (∼36 weeks' gestation) and continues into early childhood, whereas mice are born at the saccular stage and undergo rapid postnatal alveolarization between postnatal day (PN) 4 and PN14, with continued maturation through PN36. Because the neonatal murine lung closely resembles that of extremely preterm infants born at 26–32 weeks' gestation, it is a well-established model for studying bronchopulmonary dysplasia (BPD) (2, 7).

Disruption of alveolarization during this vulnerable period, particularly by exposure to supraphysiologic oxygen, leads to persistent defects in alveolar structure and function. Hyperoxia induces oxidative stress and pro-inflammatory signaling, including interleukin-1β and tumor necrosis factor-α, which impair epithelial and endothelial cell proliferation through p53- and p21-mediated cell-cycle arrest. These events inhibit secondary septal formation, prevent normal septal thinning, and disrupt epithelial–endothelial crosstalk required for microvascular integration. The resulting septal thickening, reduced alveolar surface area, and increased diffusion distance across the air–blood barrier culminate in alveolar simplification, a hallmark pathological feature underlying impaired gas exchange and long-term respiratory morbidity in BPD (8–10). Min Yee *et al*. (2009), using a hyperoxia mouse model, observed thickened elastin fiber bundles in the alveolar walls of mice exposed to ≥60% oxygen, and concluded that disrupted lung development is a primary driver of oxygen-altered lung function in children exposed to oxygen as neonates (11).

Various non-invasive and invasive ventilation strategies have been utilized to support infants with or at risk of developing BPD each with their own merits and limitations (12, 13). Non-invasive ventilation techniques, such as CPAP and nasal intermittent positive pressure ventilation (NIPPV), have gained prominence due to their potential to avoid the complications associated with intubation and mechanical ventilation (14).

A clinical trial comparing 3 approaches to the initial respiratory management of preterm neonates demonstrated neonates who received early CPAP had a 44% lower incidence of severe BPD compared to those who were treated with invasive ventilation (15% vs. 27%) (15). Similarly, a randomized trial (Nasal CPAP or intubation at birth for very preterm infants) reported that neonates managed with CPAP had a 35% reduction in the duration of oxygen therapy compared to those who underwent invasive mechanical ventilation (16).

The murine model of hyperoxia-induced lung injury closely mimics human BPD, and has been extensively used to study the effects of CPAP as a therapeutic intervention (17–20). For years, murine hyperoxia model systems have ranged oxygen concentration from 21% to 85% (21). However, the exact biological and molecular responses to CPAP remain poorly understood. This study aimed to identify a specific oxygen concentration capable of inducing disease phenotypes under hyperoxia conditions and to evaluate whether applying intermittent CPAP at the same oxygen level could ameliorate these phenotypes. Our study further explores the impact of CPAP on lung development, physiological function, and molecular pathways in the neonatal lungs. Further, the BPD phenotype established in our study was characterized through a comprehensive analysis. Our initial experiment primarily focused on histopathological, immunological, and pulmonary function outcomes in CPAP-treated and untreated cohorts.

## 2 Material and methods

### 2.1 Animal management

All mice (strain C57BL/6) experiments were performed with the approved protocol (no. 20225) from the Institutional Animal Care and Use Committee of the University of Southern California, Los Angeles, California, USA. Neonatal mice were housed with dams in temperature- and humidity-controlled rooms under a 12 h light/dark cycle, with *ad libitum* access to food and water. Litter size was standardized to minimize variability. All procedures complied with institutional animal care guidelines, and dam and litter daily monitored by animal care professionals.

### 2.2 Hyperoxia and CPAP treatment

Newborn pups (7–9 per group/experiment; depends on litter) were exposed to room air (RA) or controlled hyperoxia (60% oxygen) with or without intermittent CPAP (mentioned as CPAP in the following text) from PN1 to PN7. The hyperoxia was maintained in a hyperoxia chamber supplied with 60% medical-grade oxygen from the cylinder and was monitored daily (MiniOX I, MSA Medical). Neonatal mice assigned to the hyperoxia + CPAP group were removed daily from the hyperoxia chamber and exposed to humidified 60% oxygen delivered via continuous positive airway pressure (CPAP) using a system adapted from Reyburn *et al*. (Neonatology, 2016) (18). A soft collar (made from powder free gloves) fitted over the face and connected to a downstream manometer. Airway pressure was regulated by an adjustable leak valve positioned between the mask and manometer and continuously monitored as pressure difference. CPAP was maintained at 6 cmH₂O. CPAP was restricted to 2 h on the first day (PN1) to minimize the time separated from the nursing dam. On the following 6 days, CPAP duration was increased to 3 h/day. CPAP for neonates under RA or hyperoxia was conducted with 21% or 60% oxygen, respectively. On day 7, the pups and the nursing dam were moved to RA and allowed to grow at normal conditions (recovery) for an additional 14 days. At PN21, all the animals from the experimental groups were weighed and euthanized with Pentobarbital (200 mg/kg Intraperitoneal injection), for tissue collection, to assess histopathology, molecular markers, and differential gene expressions. The experimental groups were as follows- (1) Room air (RA)(control group); (2) Room air and CPAP (RC): RA with daily CPAP (+6 cm H_2_O, 3 h/day on days postnatal 1–7); (3) Hyperoxia (H): 60% oxygen, 24 h/day for 7 days, in a hyperoxia chamber; (4) Hyperoxia and CPAP (HC): 60% oxygen with CPAP (+6 cm H_2_O, 3 h/day for 7 days).

For the hyperoxia (50% oxygen), and CPAP (50% oxygen) groups, as shown in Supplementary Figure S1, we followed the same experimental design, setup, and procedures as described for the 60% oxygen treatment.

### 2.3 Histopathology

To explore how hyperoxia, CPAP, and their combination modulate alveolarization we measured lung MLI, and septal wall thickness of neonatal mice bred under room air (RA), room air plus CPAP (RC), hyperoxia (H, 60% O_2_), and hyperoxia plus CPAP (HC). H&E staining, mean linear intercept (MLI) quantification, and lung alveolar septa thickness were completed with a standard protocol (17, 22–24). In brief, mice were euthanized with Pentobarbital (200 mg/kg) intraperitoneal (IP) injection. The blood from the lung was cleared with PBS through ventricle perfusion, followed by lung inflation with 4% formaldehyde. The lung tissue was surgically removed and fixed in 4% formaldehyde, overnight at 4⁰C (Immersion fixation). After fixation, lungs were dehydrated through a graded ethanol series (30%, 50%, 70%, 90%, 95%, and 100%; three changes of each gradient for 30 min), followed by clearing in xylene (three changes, 30 min each). Samples were then infiltrated with molten paraffin wax (∼56 °C; 3 changes, 30 min each) and left lung lobe embedded in paraffin blocks for sectioning. The 5 μm tissue sectioning was performed with Leica Microtome. The H&E staining was performed with standard protocol, and the microscopy of H&E-stained tissue was performed with an ECHO Revolve Microscope (model, RVL-100-G). The mean linear intercept (MLI) was measured with digitally captured tissue images at 10x magnification. Six random fields were captured from each tissue section. Each section was divided into six random vertical lines and the alveolar septal intersections were counted. The MLI (µm) was calculated by the equation: the total frame size (µm) divided by the total number of counted intercepts of alveolar septa. The mean alveolar septal thickness (µm) was quantified by measuring the septal thickness in the 40X magnified images with Revolve Microscope's inbuilt image processing software. For each experimental group, around two hundred septa were individually measured, and the average value of septal thickness for each subject was used for graphical representation and statistical analysis.

### 2.4 Lung function analysis

Mice from all four exposure groups (RA, RC, H, HC) were anesthetized intraperitoneal at PN21 with a mixture of ketamine (200 mg/kg) and xylazine (20 mg/kg). 8–10 pups (littermates) were used per group for the lung function test. Anesthetized mice were placed supine on a heated surgical table and tracheostomized via a 21-gauge blunt-tip cannula, and ventilated (flexiVent, SCIREQ, Montreal, Canada) at a tidal volume of 10 mL/kg, 150 breaths/min, and PEEP value of 3 cm H_2_O. The SCIREQ FlexiVent is a computer-controlled ventilator that assesses lung mechanics using the forced oscillation technique. It delivers small, standardized pressure and volume oscillations to the respiratory system while recording airway pressure and airflow responses. As per the default flexiVent settings, three measurements (data points) were recorded concurrently for each parameter, in each subject. The respiratory system resistance, compliance, elastance and other parameters were calculated with the flexiVent software (flexiWare Version 8.0, Service Pack 4) using a 1.2-second, 2.5-Hz, single-frequency, forced-oscillation maneuver (18).

### 2.5 RNA extraction, cDNA synthesis, and real-time quantitative PCR (qPCR)

The total RNA from snap-frozen lung tissue (50 mg/sample) from all experimental groups were extracted with Quick-RNA Miniprep Plus Kit (cat # R1057, Zymo Research Corporation) per the manufacturer's instructions. The purified RNA was quantified through Thermofisher Scientific NanoDrop™ One Microvolume UV-Vis Spectrophotometer. One µg RNA per reaction was used for cDNA synthesis. The cDNA was synthesized with EasyScript Plus cDNA Synthesis Kit (cat # G236, Lamda Biotech) per the manufacturer's instructions. After completion of cDNA synthesis, the resulting reaction mixture was diluted 1:10 with DNase/RNase-free deionized water. Subsequently, 1 µL of the diluted cDNA was used as the template for each qPCR reaction. The qPCR experiment was performed with FastStart Essential DNA Green Master (cat# 06402712001, Roche), and samples were run on LightCycler® 96 System (Roche Diagnostics). Primer sequences are listed in Table 1. The qPCR data was analyzed with the software LightCycler® 96 SW 1.1.

**Table 1:** List of primers.

Genes	Forward primer (5′-3′)	Reverse primer (5′-3′)
Sftpc (spc)	ATGGACATGAGTAGCAAAGAGGT	CACGATGAGAAGGCGTTTGAG
Muc-1	CCTACCATCCTATGAGTGAATACC	GACTGCTACTGCCATTACCTG
Elastin (eln)	TTGCTGATCCTCTTGCTCAAC	GCCCCTGGATAATAGACTCCAC
TGF-β	TGGAGCAACATGTGGAACTC	GTCAGCAGCCGGTTACCA
Vegf	AGGCTGCTGTAACGATGAAG	TCTCCTATGTGCTGGCTTTG
18S rRNA	AATGGTGCTACCGGTCATTC	ACCTCTCTTACCCGCTCTCC

### 2.6 Statistical analysis

Statistical analyses were performed using GraphPad Prism version 10 (GraphPad Software, San Diego, CA, USA). Group comparisons for mean linear intercept (MLI), alveolar wall thickness, lung function parameters, and gene expression were conducted using two-way analysis of variance (ANOVA) followed by the multiple-comparison method (Tukey's test). Data are expressed as mean ± standard deviation (SD). A *p*-value of <0.05 was considered statistically significant, with significance levels indicated as *p < 0.05, **p < 0.01, ***p < 0.001, and **** *p* < 0.0001.

## 3 Results

### 3.1 CPAP maintains the alveolar architecture in hyperoxia-induced tissue damage

**Figure 1:**
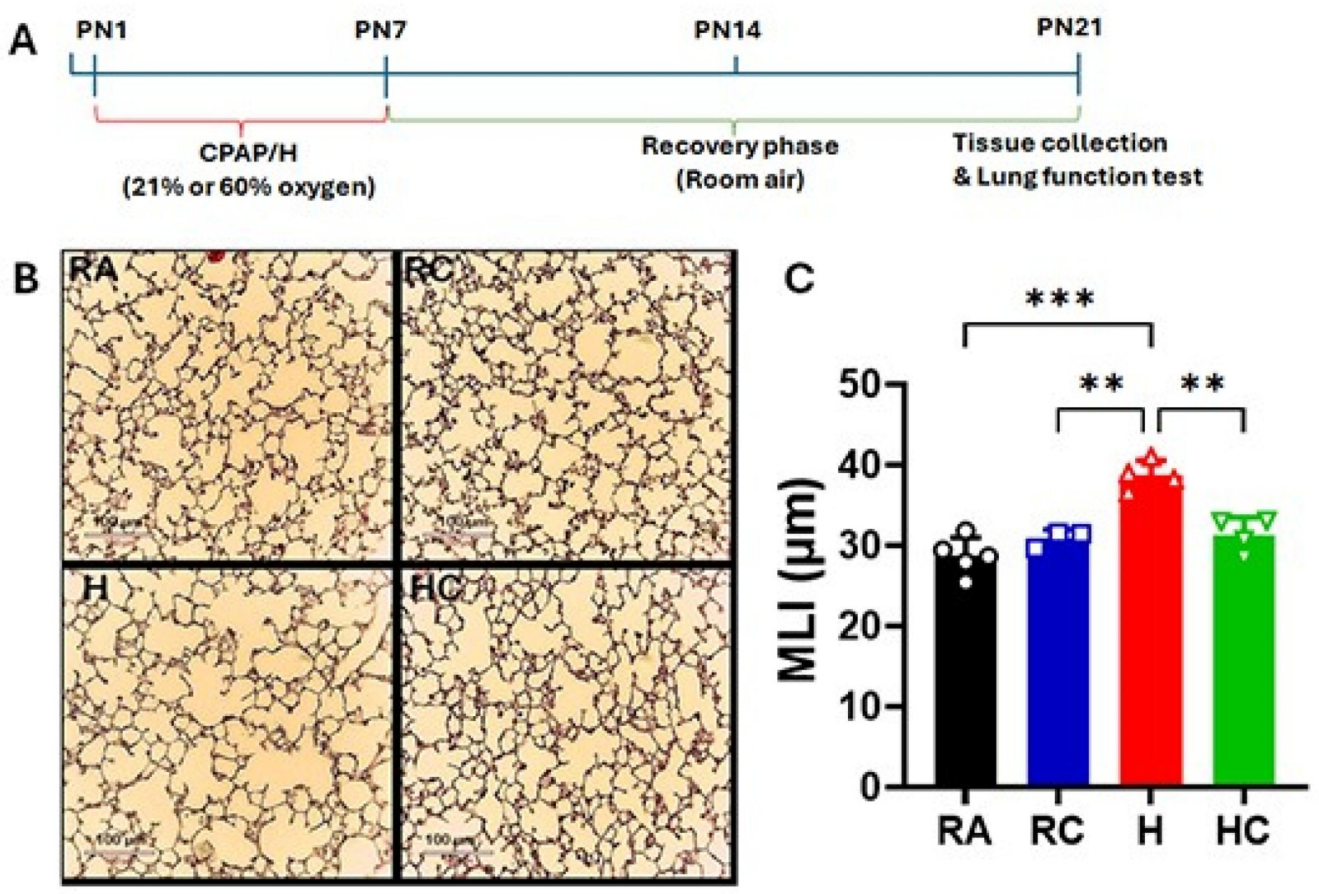
**(A)** Experimental timeline and procedure used in hyperoxia (H) and continuous positive airway pressure (CPAP) noninvasive BPD murine neonatal model. RA, Room air (21% Oxygen); RC, Room air and CPAP (21% Oxygen); H, Hyperoxia (60% Oxygen); HC, Hyperoxia and CPAP (60% Oxygen); *n* = 5-8 mice per group **(B)** Representative histological sections (H&E staining) from mouse lungs. Scale ba*r* = 100 µm. **(C)** Lung morphometry was quantified by the mean linear intercept (MLI) measurement. Data are presented as means ± SD. The statistical analysis was performed by two-way ANOVA followed by the multiple-comparison method (Tukey's test). Significance indicated in the figure: RA vs. H (***p = 0.0004), RC vs. H (**p = 0.0048), H vs. HC (**p = 0.0028).

MLI is an implied method to determine air space enlargement, including alveoli and alveolar ducts (25). Initially, exposure to 50% oxygen was used to establish the disease phenotype and evaluate CPAP effects in the hyperoxia (H) and hyperoxia-CPAP (HC) groups. This level produced a clear and biologically meaningful lung injury in the H group compared with room air (RA) controls (*p* = 0.0030), confirming effective phenotype induction. In contrast, no distinct structural modulation of alveolarization was observed between the H and HC groups (*p* = 0.8022; Supplementary Figure S1), indicating a negligible treatment effect at this exposure level. Based on these findings, the oxygen concentration was subsequently increased to 60% to further characterize the disease phenotype and evaluate the effects of hyperoxia-CPAP. As shown in Figures 1B,C, hyperoxia exposure (60% O₂, H group) caused a notable increase in mean linear intercept (MLI) to 38.72 µm compared with 28.65 µm in RA lungs (*p* = 0.0004), reflecting a substantial enlargement of alveolar spaces. Lungs subjected to CPAP under room air (RC, 30.90 µm) showed minimal change relative to RA, indicating little to no effect of CPAP in the absence of hyperoxic injury (*p* > 0.999). Interestingly, CPAP applied during hyperoxia (HC, 31.30 µm) markedly reduced the hyperoxia-induced MLI (*p* = 0.0028), demonstrating a strong protective effect against alveolar simplification.

**Figure 2:**
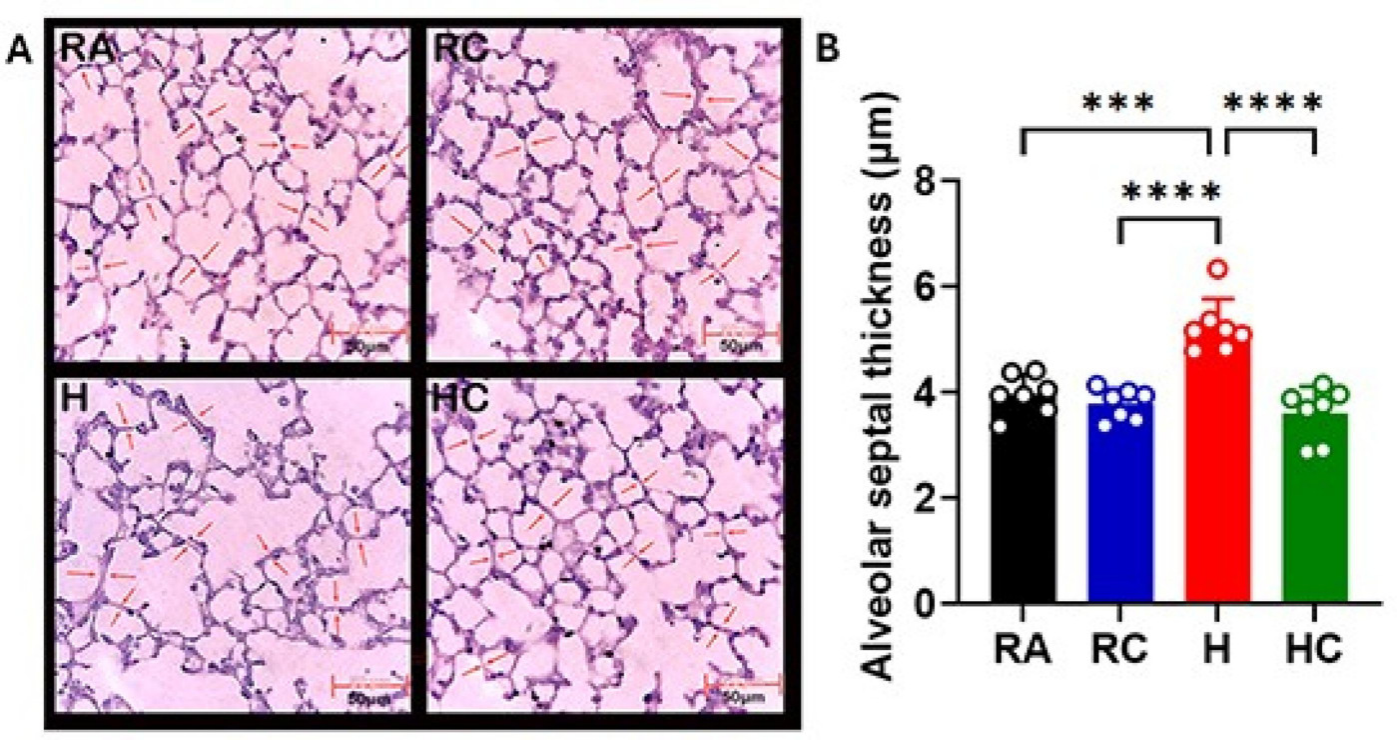
CPAP treatment maintains the pulmonary architecture. **(A)** Representative H&E images (40X magnification) for quantification of alveolar septal thickness under each condition; *n* = 5-8 mice per group. Arrows indicate the sites of measurement. **(B)** Mean alveolar septal thickness (μm) ± SD. Scale bar = 50 µm. The statistical analysis was performed by two-way ANOVA followed by the multiple-comparison method (Tukey's test). Significance indicated in the figure: RA vs. H (***p = 0.0004), RC vs. H (****p = <0.0001), H vs. HC (****p < 0.0001).

A similar trend was observed in alveolar septal wall thickness (Figures 2B,C). Hyperoxia increased septal thickness to 4.90 µm compared with 3.75 µm in RA lungs (*p* = 0.0004), representing a substantial thickening of the alveolar walls. CPAP under room air (RC, 3.74 µm) had little effect (*p* = 0.8757), whereas CPAP during hyperoxia (HC, 3.75 µm) largely prevented septal wall thickening, restoring measurements close to RA levels (*p* < 0.0001), highlighting its protective role in maintaining alveolar architecture.

### 3.2 Effect of CPAP on the pulmonary functions test

**Figure 3:**
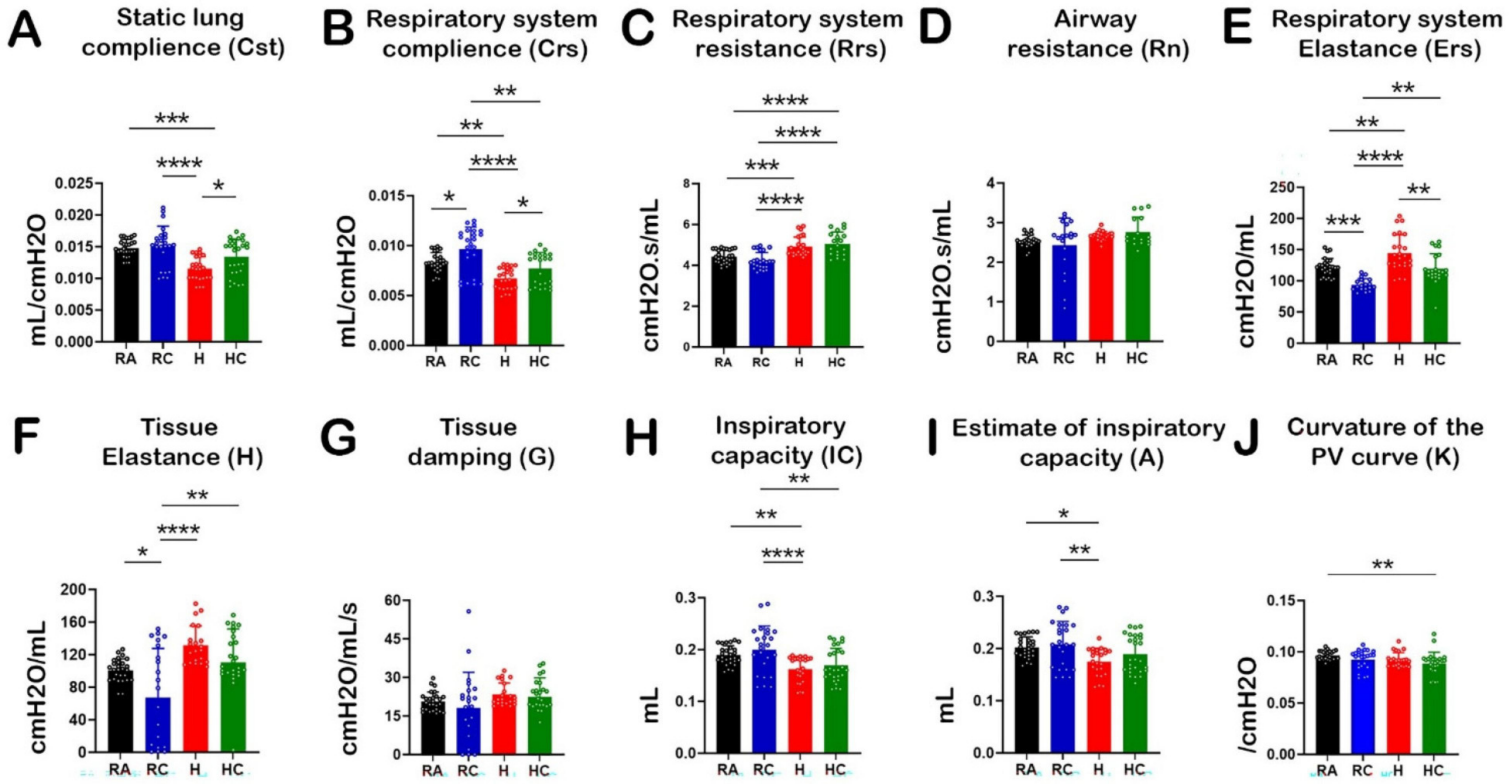
Effect of hyperoxia exposure and CPAP on respiratory system mechanics of neonatal mice. The pulmonary function parameters were recorded by the FlexiVent FX-1 module (SCIREQ, Montreal, Canada), and data were analyzed with in-build flexiVent software; *n* = 8–10 mice per group. GraphPad Prism10 was used for statistical analysis. Data are presented as means ± SD. The statistical analysis was performed by two-way ANOVA followed by the multiple-comparison method (Tukey's test). Significance indicated in the figure for **(A)** Cst: RA vs. H (***p = 0.0001), RC vs. H (****p < 0.0001), H vs. HC (*p = 0.0416). **(B)** Crs: RA vs. RC (*p = 0.0446), RA vs. H (**p < 0.0033), RC vs. H (***p < 0.0001), RC vs. HC (**p < 0.0021), H vs. HC (*p < 0.0446). **(C)** Rrs: RA vs. H (***p = 0.0009), RA vs. HC (****p < 0.0001), RC vs. H (***p < 0.0001), RC vs. HC (****p < 0.0001). **(E)** Ers: RA vs. RC (***p = 0.0004), RA vs. H (**p = 0.0044), RC vs. H (****p < 0.0001), RC vs. HC (**p = 0.0010), H vs. HC (**p = 0.0022). **(F)** H: RA vs. RC (*p = 0.0355), RC vs. H (***p < 0.0001), RC vs. HC (**p = 0.0036). **(H)** IC: RA vs. H (**p = 0.0067), RC vs. H (****p < 0.0001), RC vs. HC (**p = 0.0019). **(I)** A: RA vs. H (*p = 0.0188), RC vs. H (**p = 0.0055). **(J)** K: RC vs. HC (**p = 0.0029).

To assess the individual and combined impact of hyperoxia and CPAP on lung function, key BPD-related pulmonary parameters were evaluated across all experimental groups (Figure 3). Hyperoxia exposure (H) caused a substantial reduction in static lung compliance (Cst: 0.01153 mL/cmH₂O) compared with room air (RA: 0.01478 mL/cmH₂O; *p* = 0.0001) and CPAP-treated controls (RC: 0.01503 mL/cmH₂O; *p* < 0.0001). CPAP applied during hyperoxia (HC) partially restored Cst to near-normal levels (0.01332 mL/cmH₂O; *p* = 0.0416). Respiratory system compliance (Crs) was also markedly reduced in H (0.006702 mL/cmH₂O), with significant recovery in the HC group (0.007720 mL/cmH₂O; *p* = 0.0446). Respiratory resistance (Rrs) increased notably in H (4.907 cmH₂O·s/mL) relative to RA (4.422 cmH₂O·s/mL; *p* = 0.0009) and RC (4.260 cmH₂O·s/mL; *p* < 0.0001), whereas CPAP during hyperoxia (HC, 5.130 cmH₂O·s/mL) did not differ significantly from H (*p* = 0.6030), suggesting partial mitigation. Airway resistance (Rn) and tissue damping (G) showed minimal differences across groups (*p* > 0.05). Notably, hyperoxia caused a substantial increase in respiratory system elastance (Ers: 144.3 cmH₂O/mL) and tissue elastance, which were effectively reduced by CPAP in the HC group (119.7 cmH₂O/mL; *p* = 0.0022). Inspiratory capacity (IC) and estimated inspiratory volume (A) were diminished by hyperoxia but improved with CPAP. No differences were observed in pressure-volume curvature (K), indicating that overall lung compliance remained largely preserved across groups.

### 3.3 CPAP aids in the regulation of the expression of specific genes in hyperoxia-induced tissue damage

**Figure 4:**
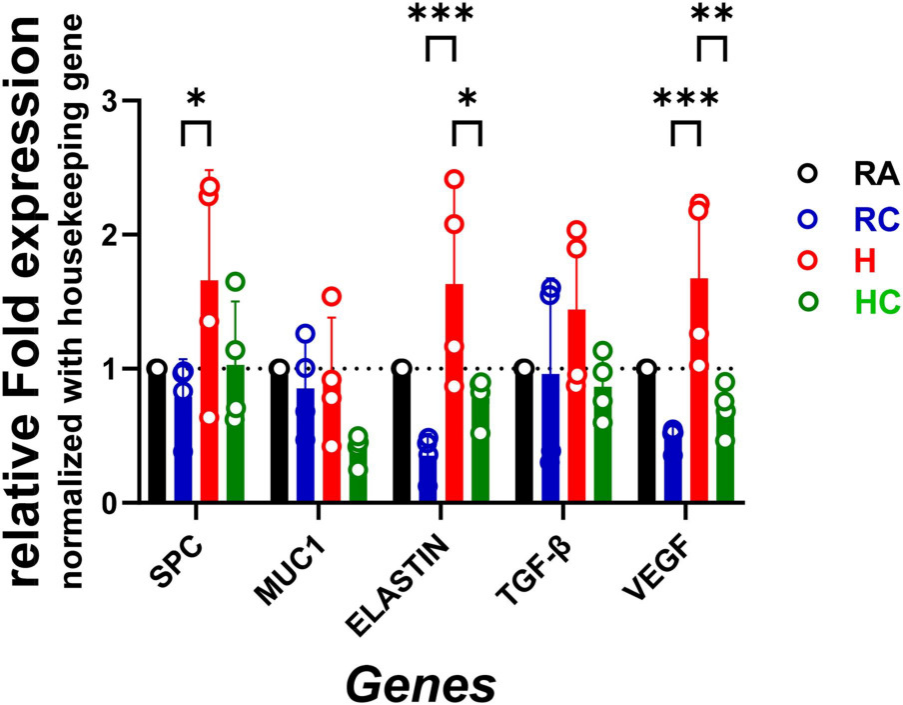
Relative mRNA expression of different genes (involved in tissue injury and repair) in the lung tissue of neonatal mice under room air and CPAP (RC), hyperoxia (H), and hyperoxia and CPAP (HC) conditions. The statistical analysis was performed by two-way ANOVA followed by the multiple-comparison method (Tukey's test). Significance indicated in the figure for SPC: RC vs. H (*p = 0.0182); for ELASTIN: RC vs. H (***p = 0.0002), H vs. HC (*p = 0.0217); for VEGF: RC vs. H (***p = 0.0006), H vs. HC (**p = 0.0066).

As shown in Figure 4, hyperoxia exposure led to a notable increase in relative fold mRNA expression of *Spc* (1.659), *elastin* (1.632), *Tgf*-β (1.440), and *Vegf* (1.674) compared with RA, indicating a substantial upregulation of genes associated with alveolar development and remodeling. CPAP under room air (RC) markedly reduced *elastin* (0.3522) and *Vegf* (0.4854) expression (*p* < 0.05), while having minimal impact on *Spc* (0.7899) and *Tgf*-β (0.9605; *p* > 0.05). Importantly, CPAP applied during hyperoxia (HC) largely restored gene expression to near-normal RA levels—*Spc* (1.029), *elastin* (0.7812; *p* = 0.0217), *Tgf*-β (0.8674), and *Vegf* (0.7016; *p* = 0.0066)—demonstrating the protective and normalizing effects of CPAP against hyperoxia-induced transcriptional changes.

## 4 Discussion

Alveolarization is critical for efficient gas exchange in the lungs. In infants with BPD, disrupted alveolarization leads to impaired lung function, resulting in poor oxygenation (26). Understanding the alveoli formation and structure is essential for developing interventions to enhance outcomes in BPD infants. A decrease in the total number of alveoli and an increase in the alveolar septa wall thickness are the two pathological hallmarks in BPD patients (21). Several cohort studies in the clinical setup have stated that nasal CPAP may be beneficial in reducing the need for intubation in preterm or low birth-weight infants (12–16, 27–29). However, the oxygen concentration (%), duration of oxygen therapy, underlying histopathology, and molecular mechanisms are still elusive. Few animal studies have explored moderate FiO_2_ levels (between 0.21 and 0.60) using clinically relevant models, but the findings suggest even these lower FiO2 may result in lung tissue damage (30). Though humans and mice follow similar stages of lung development, the alveolarization process in humans and mice is quite different (31). In humans, alveolarization begins prenatally, while in mice, this stage occurs postnatally, which makes murine models for BPD ideal. The first murine model of CPAP (21% oxygen) was developed and reported in 2015 by Catherine A. Mayer et al. to determine airway reactivity (AWR) and radial alveolar counts (RAC) in neonatal mice (32).

In our experimental model, we first evaluated 50% oxygen concentration for Hyperoxia (H) and hyperoxia-CPAP (HC). This oxygen concentration was able to induce tissue damage significantly in the H group in comparison to the room air (RA) control, but no significant difference was observed between the H and HC groups in our histopathological analysis (alveolarization pattern; Supplementary Figure S1: mean linear intercept data). Further, we increased the oxygen concentration (up to 60%) for the H and HC groups and investigated the alveolarization pattern in the lung tissue of all experimental groups. We observed a significant difference between RA vs. H, and H vs. HC in the microscopic assessment of lung architecture quantifying mean linear intercept (MLI) in histopathological slides (Figures 1A,B). A higher MLI value is an indication of the alveolar simplification (decreased number of alveoli, less surface area), a common disorder in BPD patients. We observed a significant effect of CPAP in comparison to hyperoxia alone, which was comparable to room air control. We also investigated alveolar septa wall thickness, another important parameter in alveolarization, in all experimental groups and found aligned results to the MLI (Figures 2A,B). Altogether, the result of MLI and alveolar wall thickness indicates the significant impact of CPAP on alveolarization and maintaining the lung architecture. MLI and septal wall thickness are established markers of distal airspace simplification and remodeling, respectively. While these measures do not directly address secondary septal formation or elastin localization, they do provide quantitative structural markers of alveolar simplification that are widely used in developmental lung injury models. As such, are findings support attenuation of hyperoxia-associated structural remodeling rather than full restoration of normal alveolarization. Future studies incorporating elastin and myofibroblast localization will be required to define underlying mechanisms.

Poor alveolarization influences the pulmonary growth and function in most preterm-born infants with BPD. An observational study on Pulmonary Function Tests (PFT) in very low birth weight infants disclosed a significantly higher Rrs and significantly lower Crs (33). Another report on a comprehensive PFT showed that 69% of preterm infants had impaired flow parameters, reduced compliance, and air trapping compared to term infants (34). A limited study has been conducted to see the effect of CPAP on lung function in the mouse model of BPD. In our study, we analyzed multiple lung function parameters simultaneously (Figures 3A–J). We observed the significant effect of CPAP on lung function improvement on static lung compliance (Cst), respiratory system compliance (Crs), respiratory system resistance (Ers), and estimate of inspiratory capacity(A). However, we did not observe a significant difference in respiratory system resistance (Rrs), airway resistance (Rn), tissue elastance (H), tissue damping (G), inspiratory capacity (H), and curvature of the pressure-volume curve (K).

Therapeutically, Cst is used to verify the optimal level of positive end-expiratory pressure (PEEP) (35). The decrease in compliance value is directly correlated with reduced alveolarization, lung tissue fibrosis, and interstitial changes. In our study, we also observed a significant (∼22%) decrease in Cst value in hyperoxia group subjects compared to the normoxia control, while CPAP treatment combined with hyperoxia (HC) showed a promising recovery of Cst value which was statistically comparable to normoxia control (Figure 3A). A similar pattern was observed with respiratory system compliance (Crs) measurement (Figure 3B). Respiratory system elastance (Ers) is a key parameter in assessing lung stiffness, reflecting the lung's ability to regain its original shape after being stretched. In BPD, the elevated Ers demonstrates the inability of the lungs to expand effectively during breathing, which is linked with poor alveolarization and increased respiratory effort (36, 37). In our study, CPAP improved the Ers by dropping the effect of hyperoxia alone (Figure 3E). Inspiratory capacity (IC) represents the maximum volume of air that can be inhaled after a normal exhalation while the estimate of inspiratory capacity (A) reflects the lung's ability to take in the air during inhalation. A reduction in inspiratory capacity indicates reduced lung volumes and inadequate lung expansion, which correlates with the severity of BPD (38). We also observed similar IC and A value trends in our data analysis. For both parameters (IC and A), we noticed a lower value in the hyperoxia group compared to the room air control group (Figures 3H,I). Key drivers of Rn/Rrs are airway caliber and smooth muscle tone. These were not directly visualized in the study. The histology strongly supports improved alveolar architecture with CPAP, but it does not necessarily translate to measurable effect on resistance. Such compartmental lung findings require further investigation in BPD. Though CPAP significantly reverts the effect of hyperoxia in the estimate of inspiratory capacity measurement, no significant differences were observed between the IC value of H-CPAP and the hyperoxia group alone.

The relationship between improvements in lung structure and function, and differential gene expression is critical for understanding the mechanisms of lung repair. Our histopathology and lung function data showed the promising effect of daily CPAP use combined with hyperoxic conditions. A few critical markers are TGF-β, VEGF, SP-C, elastin, fibronectin, IL-8, and MMPs, which play crucial roles in fibrosis, impaired alveolarization, abnormal lung repair, regeneration, and inflammation (39). The qPCR technique was used to detect, quantify, and analyze mRNA expression, for the selected key molecules, which are known to regulate lung structure, function, and repair mechanisms. In our moderate hyperoxia mouse model of BPD, we observed increased expression of SP-C [deficiency is linked to chronic inflammation and impaired surfactant function (40)], Elastin [dysregulation contributes to the increased airway resistance and reduced lung compliance (41)], TGF-β [associated with lung remodeling, increased smooth muscle thickness, and septal wall fibrosis (24, 42)], and VEGF [deficiency during early lung development leads to abnormal capillary development and reduced alveolar surface area (43)] in the hyperoxia group compared to room air control. In contrast to severe hyperoxia models, our moderate hyperoxia model showed elevated VEGF expression at PN21, likely reflecting a compensatory angiogenic response during recovery, consistent with previous reports of VEGF upregulation following sub-lethal or moderate lung injury (44). However, CPAP treatment was able to significantly regulate the hyperoxia-induced overexpression of these genes (Figure 4). Moreover, a detailed investigation is required to understand downstream molecular signaling at mRNA and protein levels.

From a clinical perspective, hyperoxia-induced oxidative stress and inflammation underlie BPD development, particularly in preterm infants requiring respiratory support. Early nasal CPAP, applied during the “Golden Hour,” stabilizes alveoli, maintains functional residual capacity, and reduces the need for invasive ventilation and high oxygen exposure, thereby lowering BPD risk and improving long-term pulmonary outcomes (25, 45–47). Consistent with the open lung principle in neonatal high-frequency ventilation (HFV), CPAP may promote alveolar recruitment and stabilizes lung units, preserving alveolar architecture and maintaining functional residual capacity. This structural support could translate into improved lung compliance and gas exchange, which would highlight the protective effects on both lung function and tissue integrity (48–50).

Overall, neonatal mouse outcomes (MLI, septal thickness, and lung function) are relevant to preterm human lung development because key distal lung programs—secondary septation, extracellular matrix remodeling, and microvascular expansion—are active during early postnatal life in mice and late gestation/early infancy in humans. Increased MLI reflects enlarged airspaces and reduced septation, consistent with alveolar simplification seen in “new” BPD. Increased septal thickness suggests delayed septal maturation and remodeling due to inflammation, edema, and/or ECM dysregulation, reducing gas-exchange efficiency. Corresponding abnormalities in lung mechanics provide functional evidence of impaired lung growth and persistent respiratory vulnerability.

## 5 Conclusions

Oxygen supplementation remains essential for the clinical management of BPD; however, careful titration is required to limit hyperoxia-associated lung injury. In this study, histopathological assessment confirmed that exposure to 60% oxygen reliably induced a BPD-like phenotype in neonatal mice, providing a clinically relevant model to evaluate therapeutic interventions. Using a similar oxygen concentration during intermittent CPAP therapy, we observed that CPAP promoted recovery of lung architecture by improving alveolarization, reducing alveolar septal wall thickness, and enhancing lung function in hyperoxia-exposed neonates. Overall, these findings support CPAP as a promising therapeutic approach to attenuate hyperoxia-induced lung injury and facilitate structural and functional lung repair in BPD. Ongoing longitudinal studies extending into later maturational stages (human adult-equivalent ages) will explore whether disrupted alveolarization reflects a persistently repressed state or a conditionally modifiable process, and will define the biomechanical and molecular mechanisms including epithelial progenitor dynamics, vascular–alveolar coupling, extracellular matrix remodeling, and mechanotransduction which govern developmental plasticity during lung maturation.

## 6 Limitations of this study

Despite the promising findings from this study, some limitations exist, which must be considered while translating preclinical data into the clinical setup. Human lung architecture is more complex than mice and differs in internal branching pattern (51). The hyperoxia exposure in mice does not fully simulate the multifactorial effects of BPD in preterm infants, such as mechanical ventilation, infection, and prenatal exposures. Mouse CPAP lacks precise control over pressure and flow compared to neonatal CPAP systems. This mice model does not mitigate human preterm infants' severe conditions, which are often associated with other complications like pulmonary hypertension, sepsis, and cardiovascular dysfunction. Hyperoxia and non-invasive respiratory support show promise in short-term studies, but validation of the murine model in recapitulating long-term BPD complications requires further research.

## Data Availability

The original contributions presented in the study are publicly available. This data can be found here: https://doi.org/10.6084/m9.figshare.31920057.
