# Supplementary material for: Non-invasive respiratory support attenuates lung damage in a murine model of bronchopulmonary dysplasia
Source: Front Pediatr. 2026 Apr 20;14:1755113. doi: 10.3389/fped.2026.1755113 (PMC13136091; doi:10.3389/fped.2026.1755113)
Supplement: Supplementary file 1 [file Supplementaryfile1.docx]

**Supplementary materials**

**Non-invasive respiratory support attenuates lung damage in a murine model of Bronchopulmonary Dysplasia**

Jitendra K. Tripathi, PhD^1*^; Anh Duong, BS^1^; Changgong Li, PhD^3^; Parviz Minoo, PhD^3,4^; Beiyun Zhou, PhD^5^; Inderpal Randhawa, MD^1,2^; Nathan L. Marsteller, PhD^1,2*^

1. Translational Pulmonary and Immunology Research Center, Long Beach, California, USA

2. Memorial Care Health System, Long Beach, California, USA

3. Division of Neonatology, Department of Pediatrics, University of Southern California, Los Angeles, California, USA

4. Keck School of Medicine, University of Southern California, Los Angeles, California, USA

5. Division of Pulmonary, Critical Care and Sleep Medicine, Department of Medicine, Keck School of Medicine, University of Southern California, Los Angeles, USA

* Corresponding author: nmarsteller@tpirc.org (N.L. Marsteller); jtripathi@tpirc.org (J.K. Tripathi)

**Supplementary figure**

**Figure-S1:** The bar graph shows quantification from mice lungs' histological sections (H&E staining). RA, Room air (21% Oxygen); RC, Room air and CPAP (21% Oxygen); H, Hyperoxia (50% Oxygen); HC, Hyperoxia and CPAP (50% Oxygen). Lung morphometry was quantified by the mean linear intercept (MLI) measurement. The statistical analysis was performed using GraphPad Prism version 10 (GraphPad Software, San Diego, CA, USA). Group comparisons for mean linear intercept (MLI), was conducted using two-way analysis of variance (ANOVA) followed by the multiple-comparison method (Tukey’s test). Data is expressed as mean ± standard deviation (SD). A p-value of < 0.05 was considered statistically significant, with significance levels indicated as, **p < 0.01, and ***p < 0.001.

**qPCR primer details**

| GenBank Accession | genes | Forward primer (5’-3’) | Reverse primer (5’-3’) |
| --- | --- | --- | --- |
| [NM_011359](http://www.ncbi.nlm.nih.gov/entrez/query.fcgi?cmd=Search&db=Nucleotide&term=NM_011359) | Sftpc (spc) | ATGGACATGAGTAGCAAAGAGGT | CACGATGAGAAGGCGTTTGAG |
| [NM_013605](https://www.ncbi.nlm.nih.gov/nuccore/NM_013605) | Muc-1 | CCTACCATCCTATGAGTGAATACC | GACTGCTACTGCCATTACCTG |
| [NM_007925](http://www.ncbi.nlm.nih.gov/entrez/query.fcgi?cmd=Search&db=Nucleotide&term=NM_007925) | Elastin (eln) | TTGCTGATCCTCTTGCTCAAC | GCCCCTGGATAATAGACTCCAC |
| [NM_011577](https://www.ncbi.nlm.nih.gov/nuccore/NM_011577) | TGF-β | TGGAGCAACATGTGGAACTC | GTCAGCAGCCGGTTACCA |
| NM_001025250 | Vegf | AGGCTGCTGTAACGATGAAG | TCTCCTATGTGCTGGCTTTG |
| M35283.1 | 18S rRNA | AATGGTGCTACCGGTCATTC | ACCTCTCTTACCCGCTCTCC |
